# Insulin and diet-induced changes in the ubiquitin-modified proteome of rat liver

**DOI:** 10.1371/journal.pone.0174431

**Published:** 2017-03-22

**Authors:** Shilpa R. Nagarajan, Amanda E. Brandon, Jessie A. McKenna, Harrison C. Shtein, Thinh Q. Nguyen, Eurwin Suryana, Philip Poronnik, Gregory J. Cooney, Darren N. Saunders, Andrew J. Hoy

**Affiliations:** 1 Discipline of Physiology, School of Medical Sciences & Bosch Institute, Charles Perkins Centre, University of Sydney, Sydney, NSW, Australia; 2 Diabetes and Metabolism Division, Garvan Institute of Medical Research, Darlinghurst, NSW, Australia; 3 St Vincent’s Clinical School, Faculty of Medicine, University of New South Wales, Sydney, NSW, Australia; 4 Kinghorn Cancer Centre, Garvan Institute of Medical Research, Darlinghurst, NSW, Australia; 5 School of Medical Sciences, Faculty of Medicine, University of New South Wales, Sydney, NSW, Australia; University College London, UNITED KINGDOM

## Abstract

Ubiquitin is a crucial post-translational modification regulating numerous cellular processes, but its role in metabolic disease is not well characterized. In this study, we identified the *in vivo* ubiquitin-modified proteome in rat liver and determined changes in this ubiquitome under acute insulin stimulation and high-fat and sucrose diet-induced insulin resistance. We identified 1267 ubiquitinated proteins in rat liver across diet and insulin-stimulated conditions, with 882 proteins common to all conditions. KEGG pathway analysis of these proteins identified enrichment of metabolic pathways, TCA cycle, glycolysis/gluconeogenesis, fatty acid metabolism, and carbon metabolism, with similar pathways altered by diet and insulin resistance. Thus, the rat liver ubiquitome is sensitive to diet and insulin stimulation and this is perturbed in insulin resistance.

## Introduction

The liver is exquisitely insulin-sensitive, and plays a critical role in glucose and lipid homeostasis as well as detoxification. Dysregulation of glucose and lipid metabolism in liver is a major factor in the pathogenesis of metabolic diseases including type 2 diabetes and non-alcoholic fatty liver disease (NAFLD). A primary characteristic of these metabolic diseases is the accumulation of excess lipid in the liver, known as fatty liver or hepatic steatosis [1]. This scenario is linked with impaired whole-body and hepatic insulin-stimulated glucose metabolism [2]. High-fat feeding studies with rodents demonstrates that impairment of insulin action in the liver precedes the development of insulin resistance in other glucoregulatory tissues, including skeletal muscle and adipose tissue [3, 4]. Fatty liver reduces tolerance to ischemic injury, impaired regeneration capacity [5] and increases the risk of developing hepatocellular carcinoma [6]. As such, fatty liver is an initiating factor in the development of a range of pathologies.

Transcriptomic [7], lipidomic [3], and proteomic [8] studies have provided significant insight into the mechanisms that link fatty liver and insulin resistance. Ubiquitin is a crucial post-translational modification that regulates cellular signaling in numerous processes such as metabolism, transcription, translation, vesicle transport and apoptosis [9]. Dysfunction of the ubiquitin proteasome system (UPS) is observed in multiple diseases including cancer [10] but its role in metabolic disease is relatively poorly defined. Therefore, we aimed to identify the *in vivo* ubiquitin-modified proteome (ubiquitome) in rat liver and determine changes in this ubiquitome under acute insulin stimulation and in high-fat diet-induced insulin resistance.

## Materials and methods

### Animals

All surgical and experimental procedures performed were approved by the Animal Ethics Committee (Ethics # 14/07, Garvan Institute/St. Vincent's Hospital) and were in accordance with the National Health and Medical Research Council of Australia's guidelines on animal experimentation.

Sixteen adult male Wistar rats (Animal Resources Centre, Perth, Australia) were communally housed at 22 ± 0.5°C with a controlled 12:12h light-dark cycle. Half were fed *ad libitum* a standard rodent diet (Rat Maintenance Diet; Gordons Specialty Feeds, Sydney, Australia) containing (10% fat, 69% carbohydrate, and 21% (w/w) protein) while the other half was fed a high-fat, high-sucrose diet (HFSD; 45% energy from fat, 30% energy from sucrose) made in-house [11]. After 3 weeks of feeding, indwelling catheters were implanted as described elsewhere [12].

### Insulin infusion

All animals were fasted for 5 h and randomly assigned for acute insulin stimulation or tissue collected in the basal state, eight rats in each group. Insulin (Actrapid, Novo Nordisk, Copenhagen, Denmark) was infused at a rate of 0.5 U/kg/h, and a 30% (wt/vol) variable glucose infusion was started at 4 min to maintain euglycemia, based upon [13]. In these rats, plasma samples were taken at 0, 1, 3, 5, 7.5 and 10 min for the measurement of insulin and glucose concentrations only. At 10 min, rats were euthanized with an overdose of sodium pentobarbitone (Troy Laboratories, Australia), liver rapidly dissected, freeze-clamped with aluminum tongs precooled in liquid nitrogen, and stored at −80°C for subsequent analysis. Epidydimal fat pass mass was used as an index of adiposity.

### Ubiquitome analysis

Approximately 3–4 grams of liver was lysed in a 1:4 ratio (w:vol) of tissue and lysis buffer (50 mM Tris-HCl (pH 7.5), 150 mM NaCl, 0.5% NP-40, 1 mM DTT, 1X EDTA-free protease inhibitor cocktail, 10 mM N-ethylmaleimide, 1mM sodium orthovanadate) using a POLYTRON hand homogenizer. The protein concentration was determined using a BCA assay and 500 μg of total protein was used per sample to immunopurify mono- and poly-ubiquitinated proteins using specialized ubiquitin affinity matrix (VIVAbind Ubiquitin Kit, VIVA Bioscience). After substantial washing to remove residual detergent, beads were digested for 30 min at 27°C, then reduced with 1mM DTT and left to digest overnight at room temperature with sequencing-grade trypsin (5 μg/mL, Promega), as described previously [14]. Samples were alkylated with 5mg/mL iodoacetamide and protease digestion terminated with trifluoroacetic acid. Trypsinized eluents were collected after brief centrifugation then purified and desalted using self-packed tips with 6 layers of C18 Empore disks (Pacific Laboratory Products), then dried in a SpeedVac. Samples were then resuspended in 12 μL 5% formic acid, 2% acetonitrile and stored at -80°C.

For MS analysis, 5 μL of each of the peptide samples were loaded and separated along a C18 column (400 mm, 75 μm ID, 3 μm silica beads) and introduced by nanoelectrospray into an LTQ Orbitrap Velos Pro coupled to an Easy-nLC HPLC (Thermo Fisher). Tandem mass spectrometry data was collected for the top 10 most abundant ions per scan over a 140-minute time gradient. The order of data collection was randomized to interchange between biological conditions with BSA run between each sample to minimize temporal bias.

MS/MS raw files were analyzed using MaxQuant (v1.2.7.4) [15] against the Uniprot Rat database using the Andromeda search engine integrated into MaxQuant [16]. A false discovery rate of 1% was tolerated for protein, peptide, and sites, and one missed cleavage was allowed. MaxQuant output data were filtered to remove contaminants, reverse hits, proteins only identified by site and proteins with < 2 unique peptides, and all further analysis was performed using filtered data in our *Pegasus* statistical workflow [17] (S1 Fig). An individual protein was defined as present under a particular condition if it was detected in a minimum of two replicates. Analysis of individual replicates (S2 and S3 Figs) showed a good degree of reproducibility across individual replicates within treatment groups. Protein-protein interactions and KEGG pathway analysis among the resulting protein list was analyzed using STRING (v10) [18] (with a confidence score of 0.700) and Cytoscape (v3.1.1) [19].

### Analytical methods

Blood and plasma glucose levels were determined by an immobilized glucose oxidase method (YSI 2300; Yellow Springs Instruments, Yellow Springs, OH, USA). Plasma insulin was measured by ELISA (Ultra-Sensitive Mouse ELISA, Crystal Chem, USA). Liver triacylglycerols (TAGs) were extracted using the method of Folch [20], lipids were dried under N_2_ gas, resuspended in absolute ethanol and quantified using an enzymatic colorimetric method (GPO-PAP reagent; Roche Diagnostics).

### Immunoblot analysis

Protein extraction and immunoblots from liver homogenates were performed as previously described [21]. Antibodies used were anti-phospho-Ser^473^ Akt and Atk (Cell Signaling Technology, Danvers, MA) and anti-Ubiquitin (Abcam, UK). Densitometry was performed using IPLab Gel software (Signal Analytics Corporation, Vienna, VA, USA).

### Statistical analysis

Differences among relevant groups were assessed using unpaired Student's t-test or Two-Way ANOVA using Tukey-Kramer post hoc tests as appropriate noted in figure legends. *P* < 0.05 was considered significant. Data are reported as mean ± seM.

## Results and discussion

### HFSD leads to increased adiposity, increased liver triacylglycerol content and impaired insulin-stimulated Akt phosphorylation in liver

**Fig 1:**
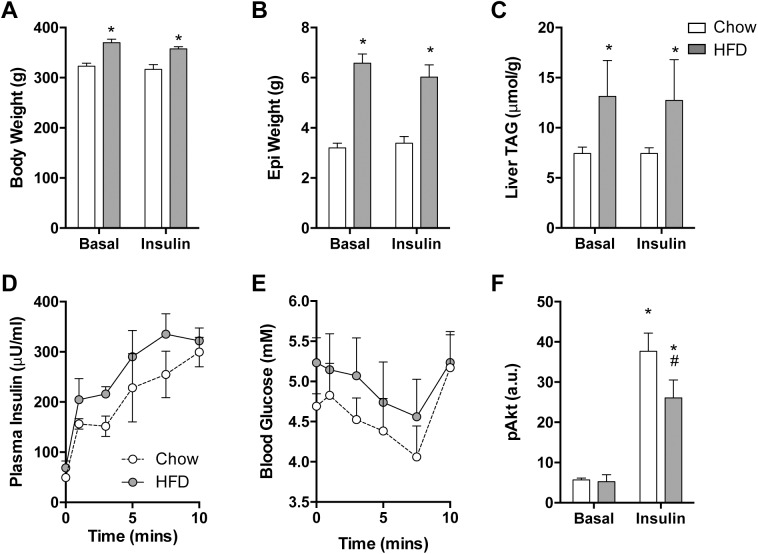
High-fat, high-sucrose feeding results in obesity and insulin resistance in male rats. **(A)** Body mass, **(B)** epidydimal fat pad mass, and **(C)** liver triacylglycerol content of rats fed a high fat diet for 4 weeks or chow control. * *P* < 0.05 vs Chow for each condition by Two-Way ANOVA followed by Tukey’s Multiple Comparisons test. **(D)** Plasma insulin and **(E)** blood glucose levels in rats infused with insulin for 10 mins at 0.5 U/kg/h. **(F)** Liver Akt phosphorylation. * *P* < 0.05 vs Basal for each condition, # *P* < 0.05 vs Chow Insulin by Two-Way ANOVA followed by Tukey’s Multiple Comparisons test. Data are mean ± SEM, n = 4.

The HFSD fed rat is a well-established model of obesity and insulin resistance [22, 23]. In this study, 4 weeks of high-fat feeding increased body mass (Fig 1A), absolute epidydimal fat pad mass (Fig 1B) and percent epidydimal fat pad mass (Chow: 3.2 ± 0.2%; HFD: 6.0 ± 0.3%; P<0.00001), and liver TAG content (Fig 1C) compared to chow-fed controls. Importantly, there were no differences in these outputs within diet groups for those animals that did or did not receive insulin stimulation (Fig 1A–1C).

Acute insulin infusion increased plasma insulin levels and at 10 mins post-infusion there was no difference between Chow and HFSD groups (Fig 1D). Further, there was no difference in blood glucose levels between groups at the end of the insulin infusion (Fig 1E). Insulin infusion increased liver Akt phosphorylation in both the chow and HFSD-fed groups but this response was blunted in the HFSD-diet group (P = 0.04; Fig 1F), indicating hepatic insulin resistance. There was no difference in total Akt levels between groups (Chow: 1.24 ± 0.23 a.u.; HFSD: 1.24 ± 0.09 a.u; P = 0.99). Calculating the ratio of phosphorylated Akt to total Akt protein levels resulted in similar findings, albeit with slightly less statistical significance (P = 0.055) as a consequence of combining the error of both measurements in a small number of animals (data not shown). Collectively, these data demonstrate the effectiveness of the HFSD diet to increase adiposity leading to a decreased response in liver for the same rise in circulating insulin.

### Global ubiquitome of rat liver

**Fig 2:**
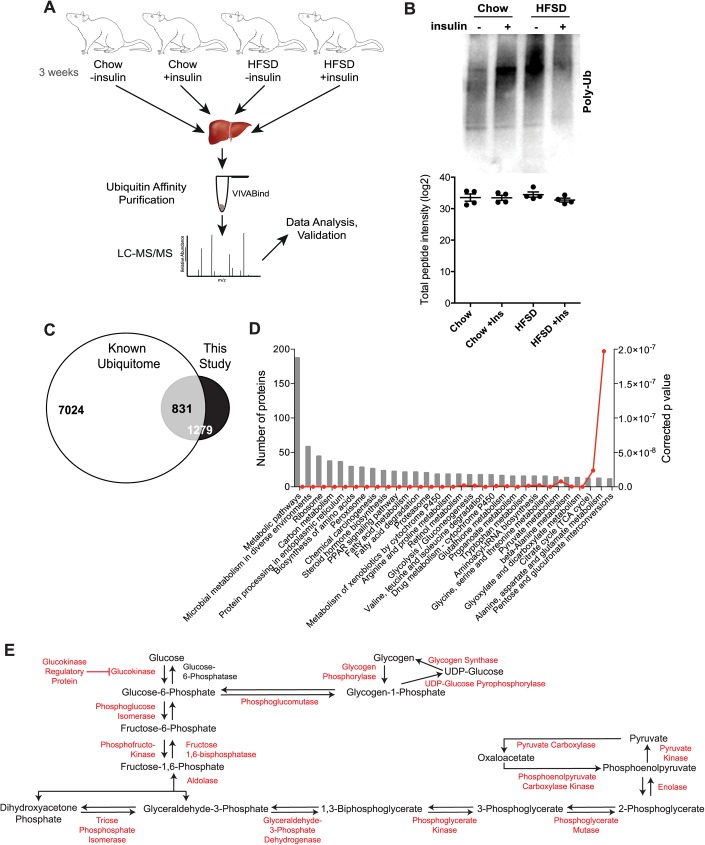
Rat liver ubiquitome. **(A)** Experimental design of the ubiquitomic analysis rat liver. **(B)** Immunoblot analysis of protein ubiquitination in representative samples of rat liver lysates. (**C)** Venn diagram of previously identified protein in this study and that in the known rat ubiquitome. **(D)** Ontology analysis of identified ubiquitinylated proteins. **(E)** Gluconeogenesis/Glycolysis biochemical pathway with ubiquitinylated enzymes marked in red.

Efforts to understand regulation of key metabolic functions of the liver to date have focused mostly on post-translational modifications such as phosphorylation [24–26] and acetylation [27, 28]. However, recent advances in affinity purification and proteomics techniques have facilitated systematic assessment of the ubiquitome *in vivo*. To more closely examine changes in the ubiquitin-modified liver proteome in rats following acute insulin stimulation and/or 4 weeks HFSD feeding, we applied AP-MS/MS using ubiquitin-affinity matrix to enrich for ubiquitinated proteins and ubiquitin binding proteins (Fig 2A). Immunoblot analysis of protein ubiquitination in representative samples of rat liver lysates showed a characteristic broad smear representing a complex mixture of ubiquitinated proteins in all conditions (Fig 2B). Label-free quantitative analysis of total peptide intensities showed no significant difference in the overall abundance of proteins isolated by ubiquitin affinity purification in each condition (Fig 2B). We identified a total of 1279 proteins following Ub-affinity purification from liver lysates across all four experimental conditions (Chow ± insulin, HFSD ± insulin). Of these, 831 proteins have been previously identified in the rat ubiquitome (total 7024; [29]). Hence, we have identified 448 new ubiquitinated proteins in rat liver (Fig 2C). Detailed lists are provided in S1 Table and a protein-protein interaction map based upon STRING analysis is provided in S2 Fig. Fig 2D shows the top 30 biological pathways represented by these proteins using ontology analysis based upon KEGG pathways. Many represent known pathways central to liver biology such as glucose, amino acid and fatty acid metabolic pathways, steroid hormone biosynthesis and drug metabolism (Fig 2D). Strikingly, we observe ubiquitination of all but 1 enzyme (glucose-6-phosphatase) involved in glycolysis/gluconeogenesis in rat liver (Fig 2E). This widespread post-translational modification suggests that ubiquitination is a key regulator of gluconeogenesis/glycolysis, acting in concert with the established transcriptional regulation of key genes (including glucose-6-phosphatate and PEPCK) [30]. Crosstalk between acetylation and ubiquitination is known to regulate the key gluconeogenesis enzyme PEPCK [31]. As our proteomics approach cannot discriminate between different ubiquitin chain topologies, validation studies beyond the scope of this work will be necessary to determine downstream functional consequences of ubiquitination (i.e. degradation via K48 chains) on regulation of signaling and metabolic pathways.

### The liver ubiquitome of rats is sensitive to insulin stimulation

**Fig 3:**
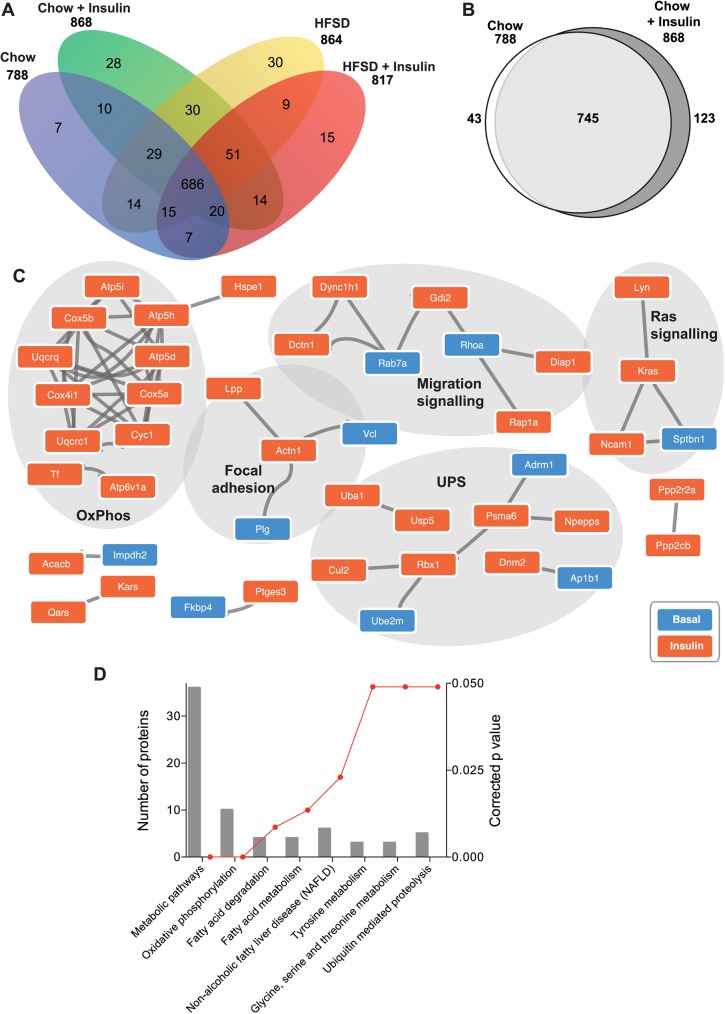
Insulin stimulation alters the ubiquitome of the rat liver. **(A)** Venn diagram of identified proteins in each group. **(B)** Venn diagram of proteins ubiquitinated in Chow compared to Chow + Insulin. (**C)** STRING analysis of differentially ubiquitinated proteins. **(D)** Ontology analysis of identified ubiquitinylated proteins.

The liver is a key insulin-sensitive tissue that contributes to the regulation of whole-body glucose homeostasis and is highly susceptible to the deleterious influences of high-fat diet [3, 4]. Of the 1279 proteins identified in this study, 788 ubiquitinated proteins were detected in chow fed animals, with 868 proteins ubiquitinated following acute insulin stimulation (Fig 3A). In HFSD rats, we identified 864 ubiquitinated proteins and 817 proteins following insulin stimulation in this group (S1 Table). Finally, we identified 686 ubiquitinated proteins in common across the 4 conditions.

To assess the potential role of the UPS in insulin action in the liver, we evaluated the specific influences of insulin on the liver ubiquitome in the chow fed rat. 745 proteins were detected in common between chow and chow + insulin conditions (Fig 3B). Of the 868 Ub-associated proteins identified in liver following i.v. insulin stimulation, 123 (14%) were unique to this condition (S2 Table), compared to the 43 (5% of 788 total) Ub-associated proteins unique to the basal state. STRING analysis of protein-protein interaction (PPI) networks within the set of differentially ubiquitinated proteins (n = 166) following insulin stimulation identified enrichment for components of OxPhos, migration signaling, focal adhesion, UPS and Ras Signaling as putative targets of ubiquitination following insulin stimulation (Fig 3C). Further, KEGG pathway analysis of these proteins identified enrichment of components of NAFLD, endocytosis, PI3K-Akt signaling and fatty acid metabolism (Fig 3D). Ubiquitination of proteins involved in fatty acid degradation and metabolism following insulin stimulation suggests a novel mechanism contributing to insulin inhibition of fatty acid catabolism (i.e. beta oxidation) and stimulation of fatty acid synthesis [32].

Targeted analysis of protein ubiquitination has demonstrated that insulin stimulation leads to ubiquitination of a range of key insulin signaling intermediates which acts as a negative feedback mechanism. For example, acute insulin stimulation of FAO hepatocyte cells increased IRS-2 ubiquitination and lowered IRS-2 protein levels [33]. Conversely, insulin stimulation of primary rat hepatocytes reportedly reduced protein ubiquitination [34]. Insulin stimulation of mouse hepatocytes expressing HA-tagged ubiquitination increased APPL1 ubiquitination in a TRAF6-mediated mechanism [35]. Our systematic approach to identify the insulin-sensitive rat liver ubiquitome did not detect IRS-1, IRS-2 or APPL1, possibly reflecting sensitivity limitations or differences between *in vitro* and *in vivo* models. Collectively, we have identified a broad range of liver proteins with altered ubiquitination in response to acute *in vivo* insulin stimulation. These will provide an important basis for ongoing targeted studies to better understand regulatory mechanisms underpinning insulin action in the liver.

### The rat liver ubiquitome is sensitive to high-fat diet

**Fig 4:**
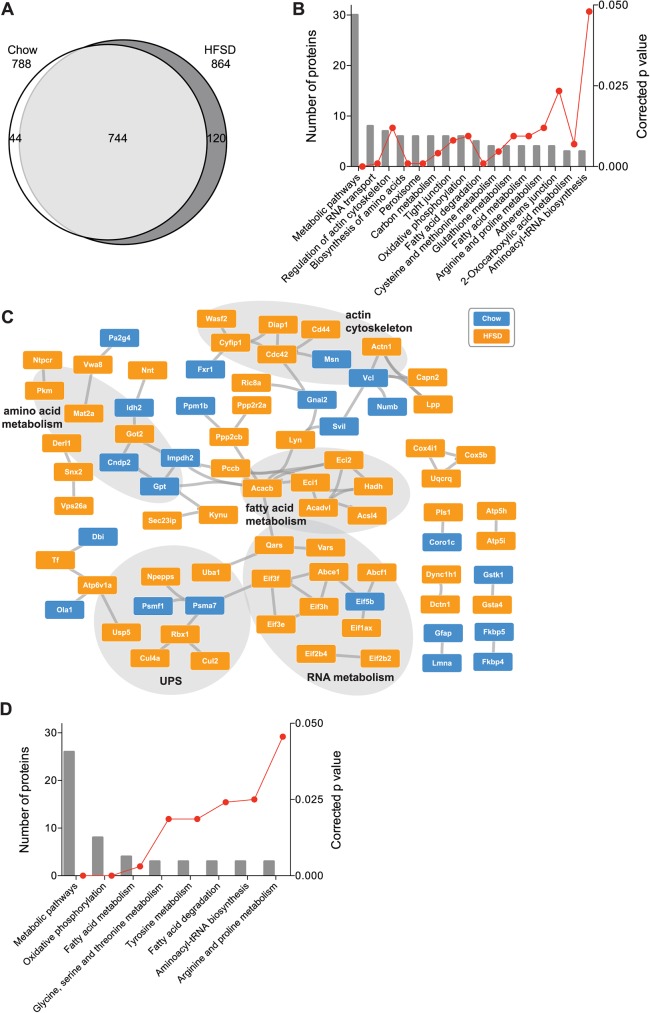
High-fat, high-sucrose diet alters the ubiquitome of the rat liver. **(A)** Venn diagram of identified proteins ubiquitinated in HFSD compared to HFSD + Insulin. **(B)** Ontology analysis of identified ubiquitinylated proteins in HFSD compared to HFSD + Insulin. **(C)** STRING analysis of differentially ubiquitinated proteins. **(D)**. Ontology analysis of identified ubiquitinated proteins responsive to insulin in Chow but not in HFSD.

The liver is highly susceptible to the deleterious influences of high-fat diet, including dysregulated glucose and lipid homeostasis [3, 4]. Altered signal transduction, including via differential protein phosphorylation, are proposed to underpin these systemic phenotypes [36]. However, other post-translational modifications such as ubiquitin are also likely to be important regulators of signaling and metabolic pathways as a consequence of HFSD feeding. We observed 744 ubiquitinated proteins in common between chow and HFSD animals (Fig 4A). Of the 864 ubiquitinated proteins identified in HFSD, 120 (14%) proteins were unique to the HFSD group. This compared to 44 (6%) of the 788 ubiquitinated proteins unique to chow-fed animals (Fig 4A). Analysis of KEGG pathways and PPI networks within this set of differentially ubiquitinated proteins identified enrichment of proteins involved in RNA metabolism, actin cytoskeleton, and amino acid and fatty acid metabolism (Fig 4B and 4C).

These diet-induced changes in the liver ubiquitome differ from observations made following exposure of HepG2 hepatocellular carcinoma cells to single fatty acids *in vitro*. For example, ubiquitin levels in HepG2 cells (detected by western blot) were not altered by exposure to the polyunsaturated fatty acid linoleate with or without the addition of exogenous FABP4 [37], which is increased in the plasma of obese patients [38]. Conversely, exposing HepG2 cells to the saturated fatty acid palmitate did lead to ubiquitination of IR, IRS-1 and Akt [39]. Palmitate does have significant effects on cell biology, but interestingly many of these effects are lost with the addition of other unsaturated fatty acids such as linoleate or oleate [40]. Further, the use of single fatty acids to increase extracellular lipid levels does not model the environment of HFSD [41].

We have performed similar analyses of the binary comparisons between HFSD vs HFSD + Insulin (S5 Fig), and Chow + Insulin vs HFSD + Insulin (S6 Fig). Briefly, 56 proteins were uniquely ubiquitinated in the HFSD + Insulin group compared to the HFSD group, whereas 103 were identified exclusively in the HFSD group compared to HFSD + Insulin (S5A Fig). Additionally, only 46 proteins were uniquely identified in the HFSD + Insulin group compared to the Chow + Insulin groups, whilst 97 proteins were only ubiquitinated in the Chow + Insulin group (S6A Fig). These proteins are involved in a diverse range of metabolic pathways (S5B and S6B Fig).

Insulin resistance is defined as a blunted response by cells to insulin stimulation. E3 ubiquitin ligases have been implicated in the pathogenesis of insulin resistance by modulating insulin signaling (see review [42]). Much of the data underlying this model comes from targeted analysis of ubiquitination of individual insulin signaling intermediates [43, 44], rather than the systematic analysis of global changes in ubiquitome in pre-clinical models of insulin resistance presented here. Our dataset provided a unique opportunity to characterize changes in the liver ubiquitome associated with insulin resistance. We identified 310 ubiquitin modified proteins that were responsive to insulin stimulation in chow or HFSD animals (S3 Table). 99 (40%) of these 310 proteins responded to insulin stimulation in the chow group but failed to respond in the HFSD group. Notably, these proteins are involved in oxidative phosphorylation and fatty acid metabolism (Fig 4D), highlighting these pathways as key targets of ubiquitin in insulin resistance. For example, one of these fatty acid metabolism proteins was ACSL4 which is increased in patients with NAFLD compared to controls [45]. Further, we identified altered ubiquitination of subunits of complex 2 and 5 of the electron transport chain, which is consistent with a role for altered oxidative phosphorylation in the development of liver insulin resistance [46]. Conversely, 89 proteins (36%) responded to insulin stimulation in the HFSD group but not in the chow group, with 61 proteins (24%) responding to insulin in both groups. Together with our identification of ubiquitination of all but one of the enzymes in glycolytic/gluconeogenic pathways, these novel data suggest that ubiquitination is a key regulator of the pathogenesis of liver insulin resistance and may play a role in fatty liver associated diseases.

## Conclusion

Herein, we have described the systematic characterization of the rat liver ubiquitome and report the effects of acute *in vivo* insulin stimulation and high-fat, high-sucrose diet. Specifically, we observed ubiquitination of proteins involved in key metabolic pathways, in particular gluconeogenesis/glycolysis, oxidative phosphorylation and fatty acid metabolism. Hence, ubiquitination is likely a novel mechanism acting at multiple levels to regulate whole-body euglycemia and lipidemia. Further, widespread changes in the ubiquitin modified proteome may mediate the pathogenesis of fatty acid-associated diseases.

## Supporting information

S1 Fig Statistical workflow. Flow diagram showing data analysis method from mass spectrometry raw data.
(TIF)

S2 Fig Proteomics data variability analysis. **(A)** Histograms showing peptide abundance distributions in individual replicates for each condition. **(B)** Multiple regression analysis of peptide abundance in individual replicates.
(TIF)

S3 Fig Venn analysis of proteins identified in each replicate. (TIF)

S4 Fig Protein-protein interactome of the total liver ubiquitome. (TIF)

S5 Fig Insulin stimulation alters the hfsd ubiquitome of the rat liver. **(A)** Venn diagram of proteins ubiquitinated in High-Fat Sucrose Diet (HFSD) compared to HFSD + Insulin. **(B)** Ontology analysis of identified ubiquitinylated proteins. (**C)** STRING analysis of differentially ubiquitinated proteins.
(TIF)

S6 Fig Effects of diet on insulin stimulated ubiquitome of the rat liver. **(A)** Venn diagram of proteins ubiquitinated in Chow + Insulin compared to High-Fat Sucrose Diet (HFSD) + Insulin. **(B)** Ontology analysis of identified ubiquitinylated proteins. (**C)** STRING analysis of differentially ubiquitinated proteins.
(TIF)

S1 Table Proteins identified in the rat liver ubiquitome for each condition. (XLSX)

S2 Table Ubiquitinated proteins unique to each condition. (XLSX)

S3 Table Proteins ubiquitinated in response to insulin. (XLSX)
