# Correction: Insulin and diet-induced changes in the ubiquitin-modified proteome of rat liver

**DOI:** 10.1371/journal.pone.0184610

**Published:** 2017-09-08

**Authors:** Shilpa R. Nagarajan, Amanda E. Brandon, Jessie A. McKenna, Harrison C. Shtein, Thinh Q. Nguyen, Eurwin Suryana, Philip Poronnik, Gregory J. Cooney, Darren N. Saunders, Andrew J. Hoy

There is an error in S1 Table in the Supporting Information files. The worksheet "Chow" listing the proteins that were identified in Chow-fed animals is a duplicate of the worksheet "HFD". Please view the complete, correct S1 Table below.

## Supporting information

S1 Table Proteins identified in the rat liver ubiquitome for each condition. (XLSX)
