# Supplementary figures and images for: Insulin and diet-induced changes in the ubiquitin-modified proteome of rat liver
Source: PLoS One. 2017 Mar 22;12(3):e0174431. doi: 10.1371/journal.pone.0174431 (PMC5362237; doi:10.1371/journal.pone.0174431)

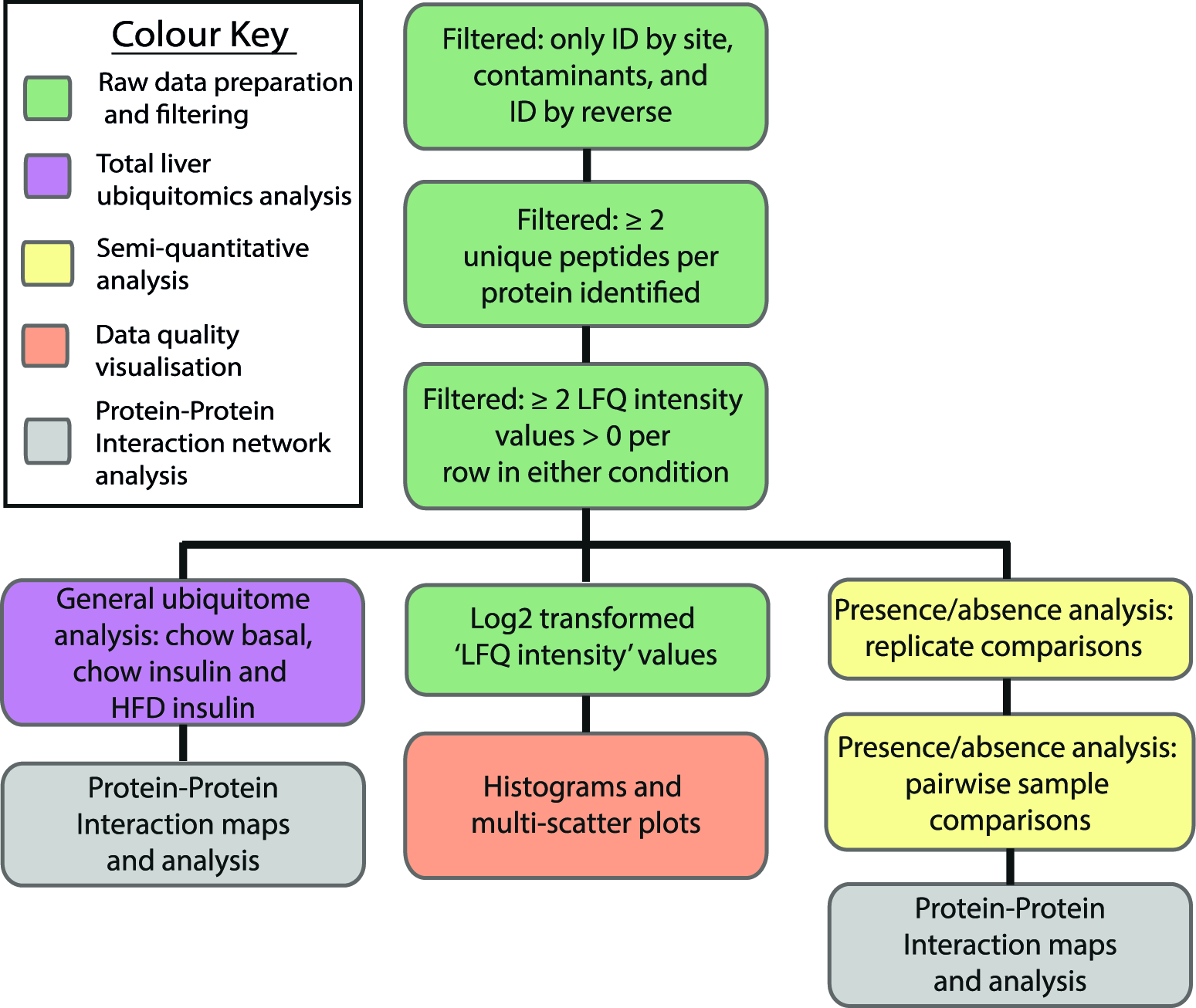

Supplement: S1 Fig — Flow diagram showing data analysis method from mass spectrometry raw data. (TIF) [file pone.0174431.s001.tif]

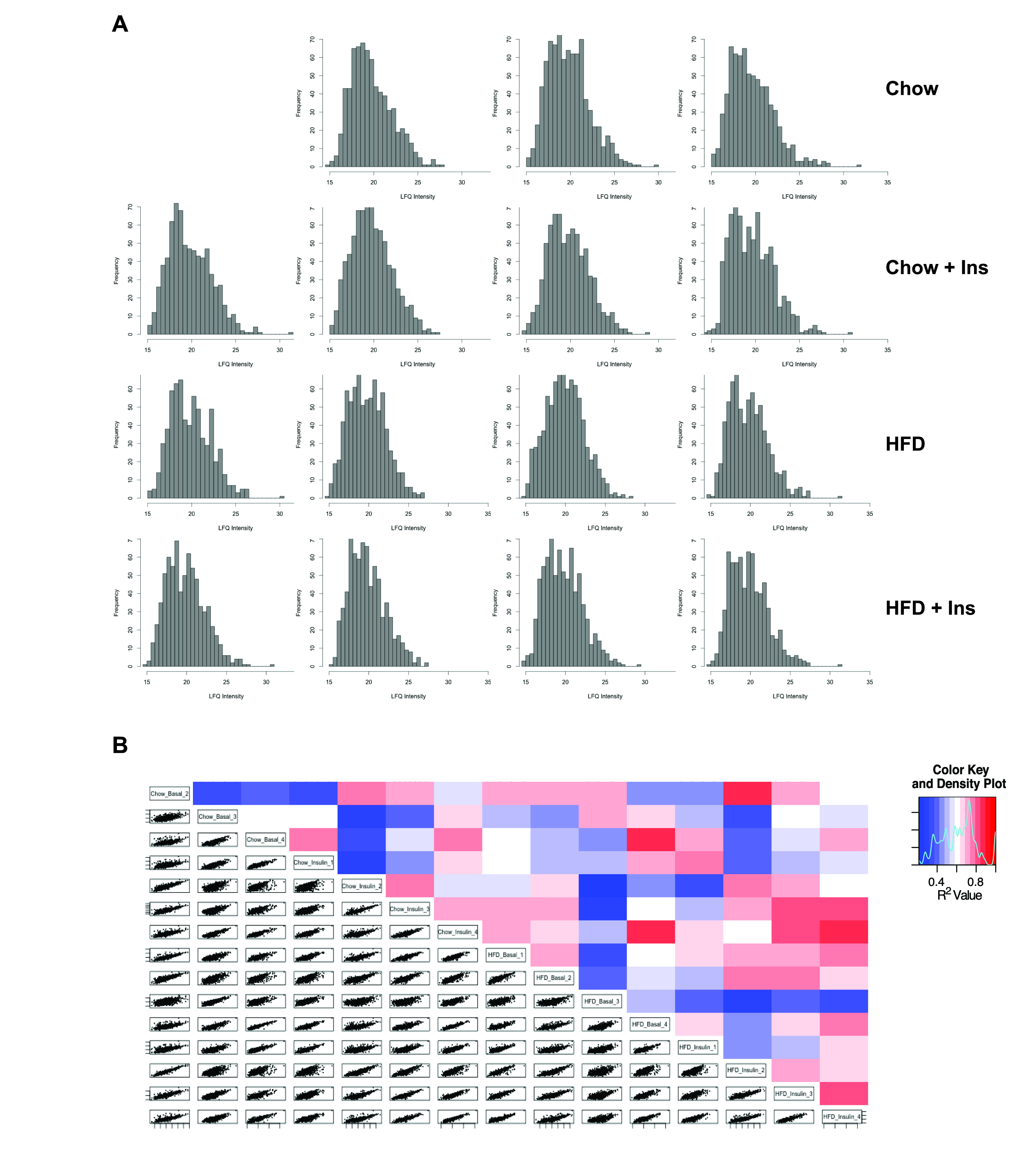

Supplement: S2 Fig — (A) Histograms showing peptide abundance distributions in individual replicates for each condition. (B) Multiple regression analysis of peptide abundance in individual replicates. (TIF) [file pone.0174431.s002.tif]

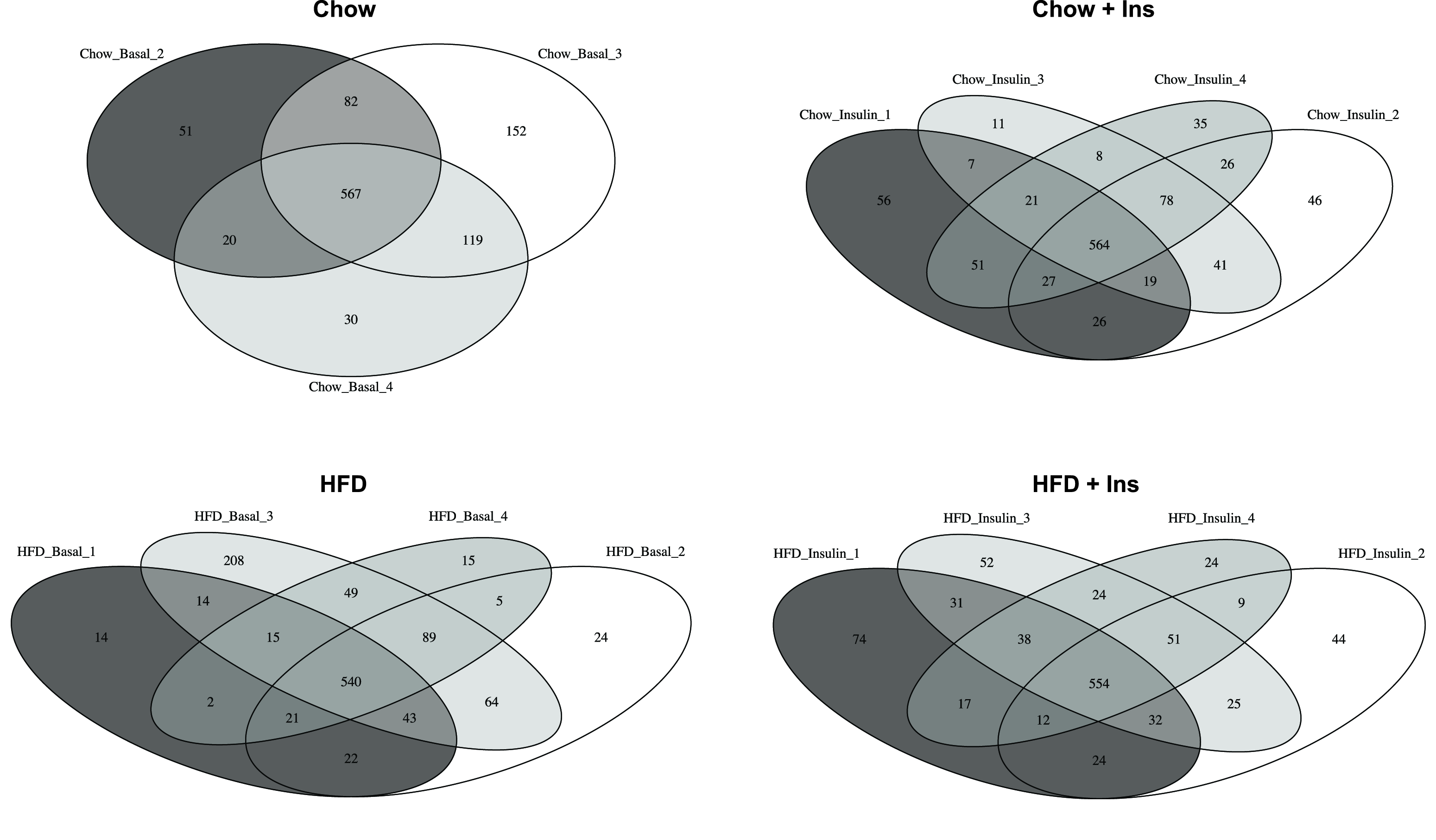

Supplement: S3 Fig — (TIF) [file pone.0174431.s003.tif]

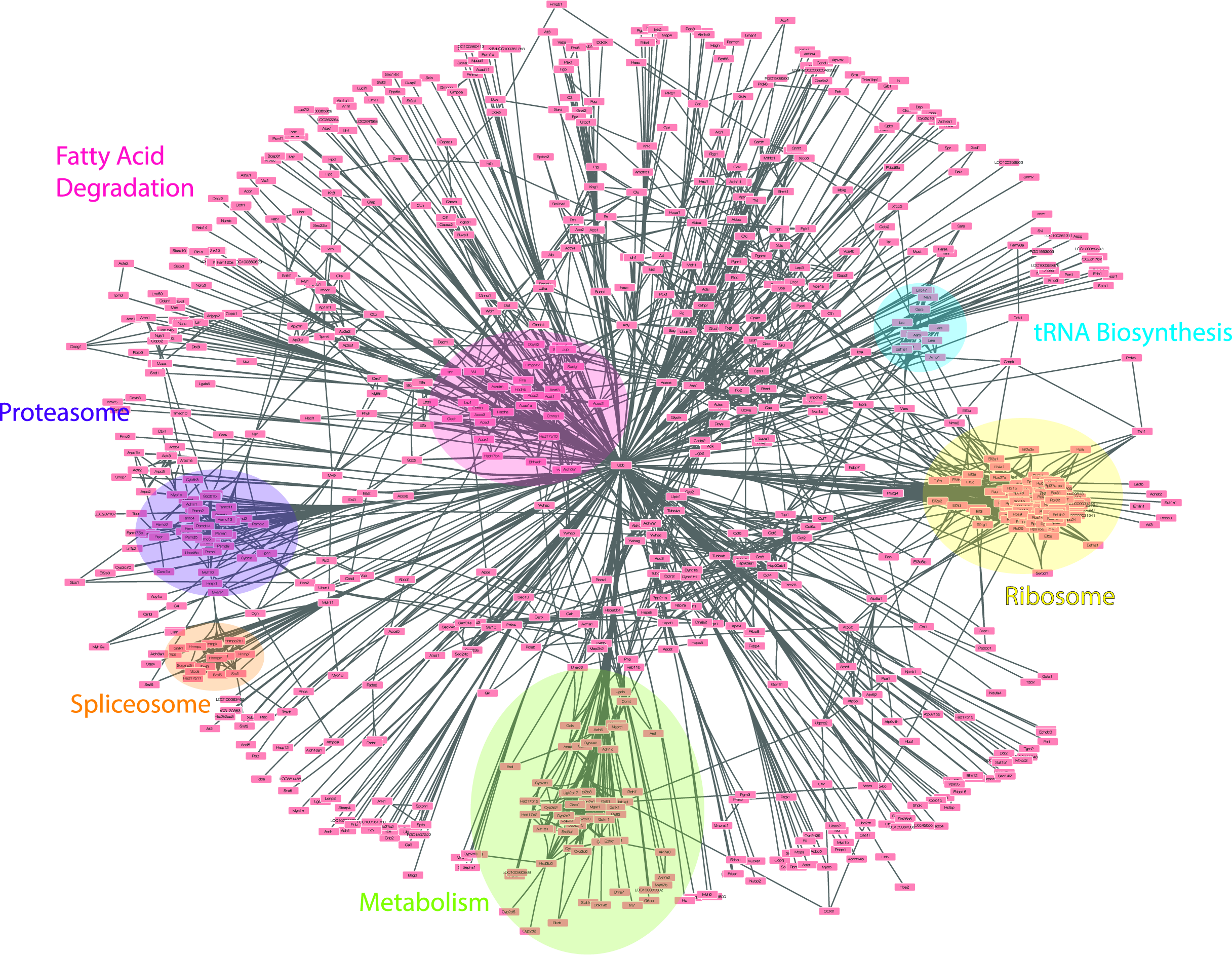

Supplement: S4 Fig — (TIF) [file pone.0174431.s004.tif]

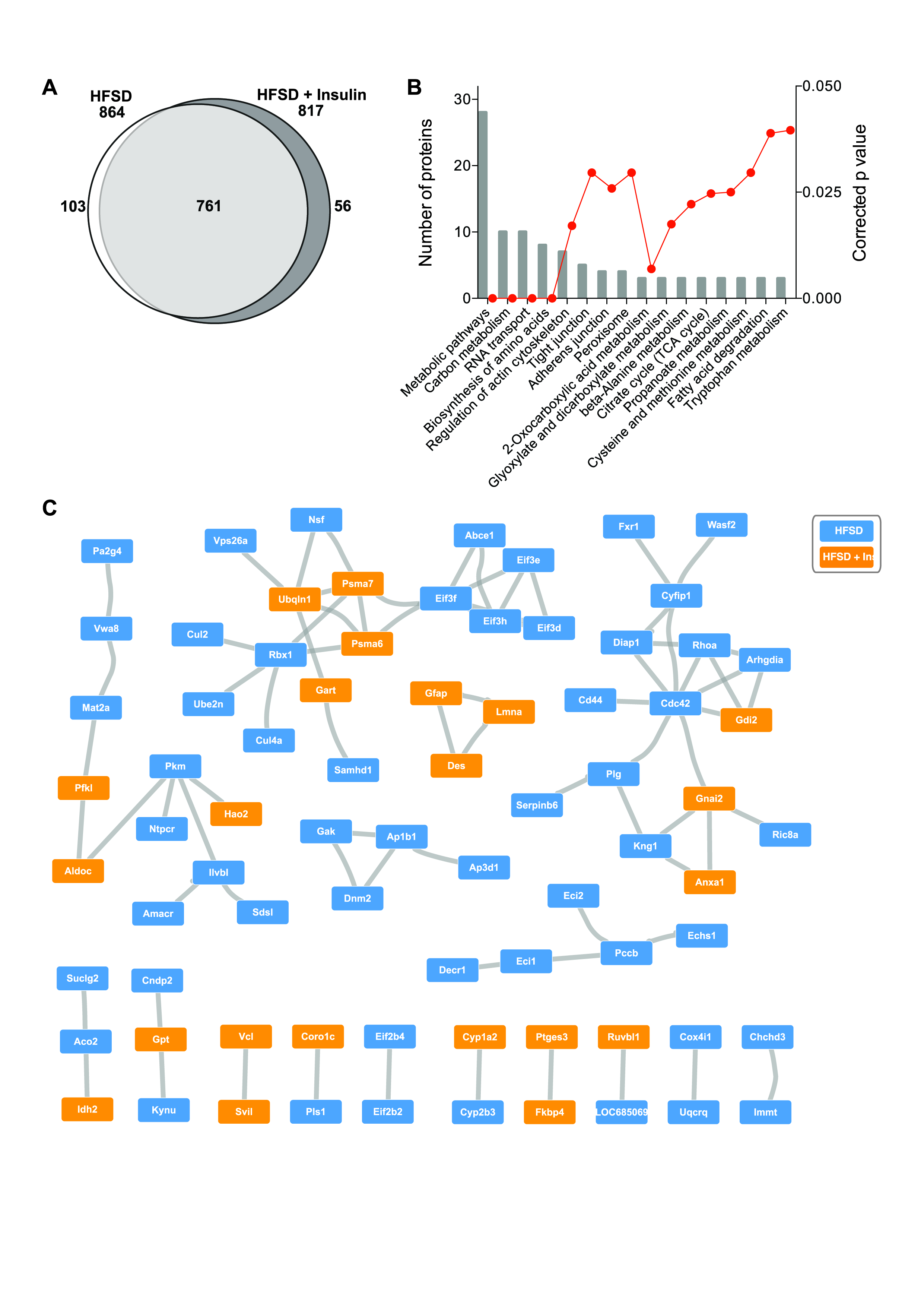

Supplement: S5 Fig — (A) Venn diagram of proteins ubiquitinated in High-Fat Sucrose Diet (HFSD) compared to HFSD + Insulin. (B) Ontology analysis of identified ubiquitinylated proteins. (C) STRING analysis of differentially ubiquitinated proteins. (TIF) [file pone.0174431.s005.tif]

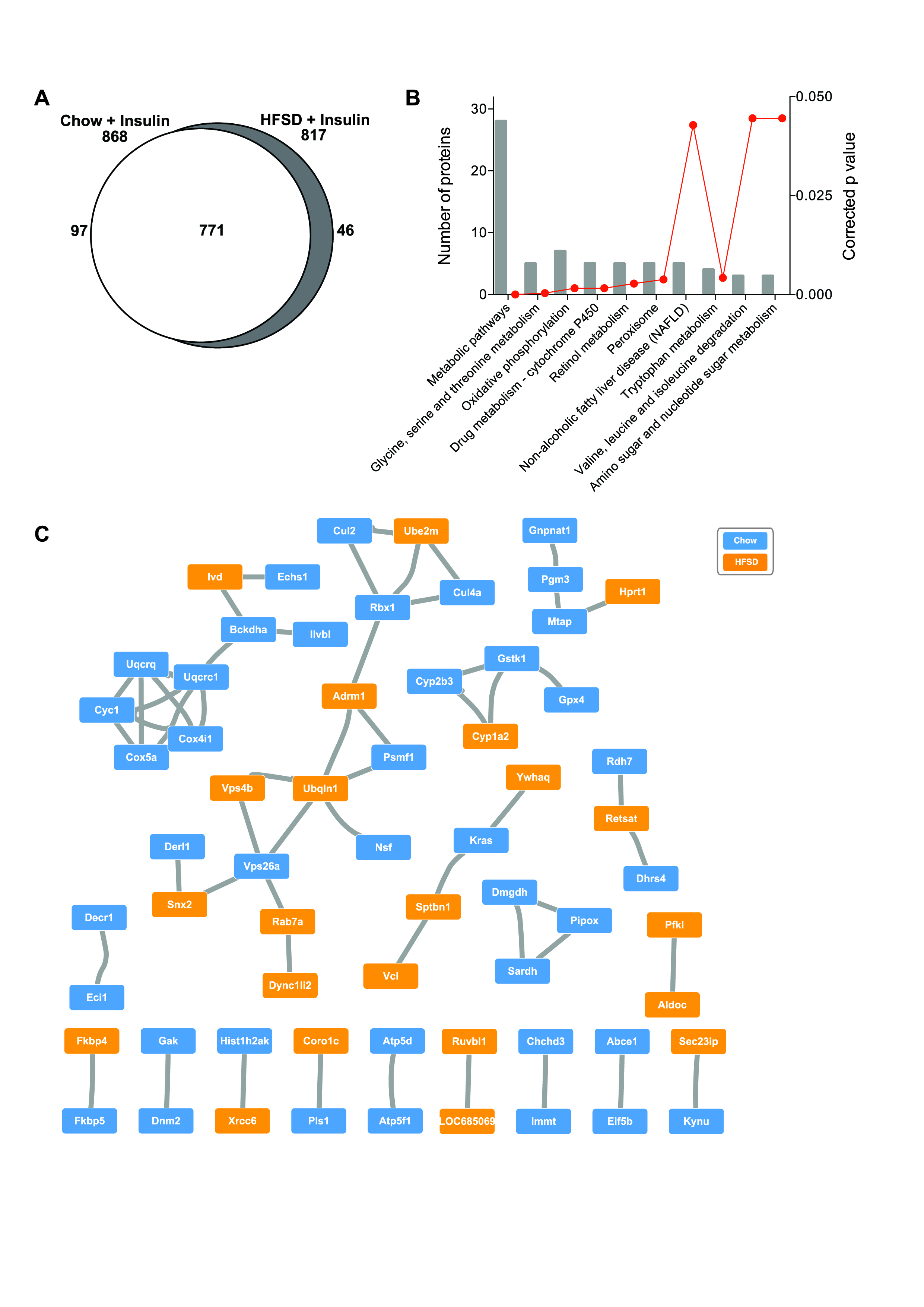

Supplement: S6 Fig — (A) Venn diagram of proteins ubiquitinated in Chow + Insulin compared to High-Fat Sucrose Diet (HFSD) + Insulin. (B) Ontology analysis of identified ubiquitinylated proteins. (C) STRING analysis of differentially ubiquitinated proteins. (TIF) [file pone.0174431.s006.tif]
